# Engaging With a Wiki Related to Knowledge Translation: A Survey of WhatisKT Wiki Users

**DOI:** 10.2196/jmir.3001

**Published:** 2014-01-21

**Authors:** Deepa Mathew, K Ann McKibbon, Cynthia Lokker, Heather Colquhoun

**Affiliations:** ^1^Health Information Research UnitDepartment of Clinical Epidemiology and BiostatisticsMcMaster UniversityHamilton, ONCanada; ^2^Centre for Practice-Changing Research (CPRC)Ottawa Hospital Research InstituteOttawa Hospital – General CampusOttawa, ONCanada

**Keywords:** knowledge translation, wiki, usability

## Abstract

**Background:**

In 2008, WhatisKT wiki was launched as a collaborative platform for knowledge translation (KT) researchers and stakeholders to debate the use and definitions of KT-related terms. The wiki has definitions for over 110 terms from disciplines including health care, information technology, education, accounting, and business. WhatisKT wiki has over 115 registered users. Approximately 73,000 unique visitors have visited the wiki since 2008. Despite annual increases in visitors and regular maintenance of the wiki, no visitors have contributed content or started a discussion.

**Objective:**

We surveyed wiki users to gain an understanding of the perceived value of the website, reasons for not engaging in the wiki, and suggestions to facilitate collaboration and improve the usability of the wiki.

**Methods:**

We surveyed three cohorts: KT Canada members who were previously invited to join the wiki, registered wiki members, and unregistered visitors. The first two cohorts completed a Web-based survey that included the System Usability Scale (SUS) questionnaire to assess usability; additionally 3 participants were interviewed. Unregistered wiki visitors were surveyed with polls posted on the wiki. The study received ethics approval from the McMaster University Faculty of Health Sciences Research Ethics Board.

**Results:**

Twenty-three participants completed the Web-based and SUS surveys; 15 participants indicated that they would collaborate on the wiki. The mean SUS score of 67 (95% CI 56-77) indicated that the wiki could be considered for design improvements. Study participants indicated that the wiki could be improved by email notification regarding new terms, better grouping of terms, user friendly interface, and training for users interested in editing content.

**Conclusions:**

The findings from this survey will be used to enhance the design and content of WhatisKT wiki. Further feedback from participants will be used to make the wiki an ideal collaboration platform for KT researchers interested in terminology.

## Introduction

### Knowledge Translation

Interest in knowledge translation (KT) has increased considerably in the past several years. Research communities use over 100 KT terms worldwide. Terms such as knowledge to action, knowledge transfer, knowledge exchange, research utilization, implementation, quality improvement, dissemination, and diffusion are often used by stakeholders in the field. A relatively widely used definition of KT was developed by the Canadian Institutes of Health Research (CIHR), which defines KT as “the exchange, synthesis and ethically-sound application of knowledge—within a complex system of interactions among researchers and users—to accelerate the capture of the benefits of research for Canadians through improved health, more effective services and products, and a strengthened health care system” [1]. However, even health care funding agencies vary in the operational definition of KT [2].

The existence of multiple terms and definitions related to KT is a challenge for researchers trying to identify previous research and for communicating with others. Despite the increase in popularity of KT research, finding meaningful and consistent definitions for KT terms is a challenge [3]. McKibbon and colleagues reviewed over 2600 articles published in health care journals and identified 100 individual terms to describe KT research and a lack of consistency in the use of terms [4].

### Wikis

Wikis are an online, Web-based, collaborative platform where anyone with proper access rights can modify content and contribute to online discussions. The most well-known wiki is Wikipedia. Wikis facilitate ease of documentation, learning, and ad-hoc collaboration, thereby developing online communities of practice and user groups, regardless of organizational affiliation [5]. Wikis have been used for collaborative content development for clinical decision support [6], and by patients contributing to the development of clinical practice guidelines [7] and providing input in developing care plans [8]. Some studies have researched how wikis are or could be used in health care, such as the intention of physicians to use social media including wikis to share medical content [9] and emergency care professionals’ beliefs around the use of a wiki to share reminders that promote best practices in trauma [10].

**Figure 1:**
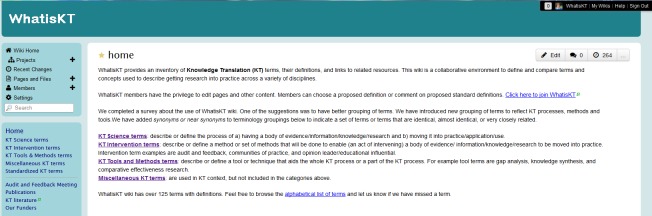
Screenshot of WhatisKT.

In 2008, WhatisKT wiki was launched as a collaborative platform for KT researchers and stakeholders to debate the use and definitions of KT-related terms (Figure 1). The wiki has over 110 terms with definitions from a variety of disciplines including health care, social sciences, information technology, education, accounting, and business. Each term has a dedicated page with a short description of definitions and the discipline where the term originated. In 2011, in an effort to generate discussion around terms and definitions, wiki organizers analyzed definitions for the 13 most frequently visited terms and, by a process of discussion and consensus, 2 team members selected one definition as being representative of that term. The definitions were selected based on having the most of 12 identified concepts within the CIHR definition of KT, clarity, comprehensiveness, reputation of source, and breadth of coverage [11]. The preferred published definition was presented at the top of the list of definitions on the wiki page for that term and highlighted. In September 2012, to provide better clarity and structure to the wiki, all terms were grouped into “Core KT” terms (eg, implementation research, quality improvement, research utilization) and “Additional KT” terms (eg, adoption, best practice, change). Core terms directly associate with the broad field of KT and moving knowledge into practice. Additional KT terms are those more specific terms used in the KT context but not representative of the field. The 13 terms with standardized definitions were grouped under “Standardized KT” terms (eg, research utilization, knowledge mobilization, diffusion of innovation). The wiki’s navigation bar facilitates access to core, standardized, and additional terms. The wiki also has links to sources of KT publications and literature.

Almost 73,000 unique visitors have visited the wiki since 2008. There are over 115 registered members. The average number of visitors per month to the wiki has increased steadily from about 196 in 2008, to 1900 in 2012 [11], and more than 2400 per month in 2013. The percentage of returning visitors also increased from less than 3% in 2008 to over 10% in 2012 [11]. The organizers regularly update the wiki, and the most recent changes are readily available for viewing by all wiki visitors. Despite the increase in visitors and regular maintenance of the wiki, no member has contributed source material or started a discussion on the wiki.

### Usability

Usability can apply to any system or product with which a user interacts. Usability is defined as the “extent to which a product can be used by specific users to achieve specified goals with effectiveness, efficiency and satisfaction in a specified context of use” [12] and depends on factors such as the product design, ease with which users can learn the product, efficiency of the product in helping users achieve their objective, ease with which users can memorize product features for future use, and user satisfaction. Usability tests allow quantification of how well a product satisfies the user’s needs. In health care systems, usability studies can reveal use-related hazards [13]. Usability evaluations are usually conducted by experts and provide subjective data regarding user satisfaction. Surveys and questionnaires, interviews, focus groups, and user observation can be used to gather a range of usability information. The System Usability Scale (SUS) is a 10-item, 5-point Likert scale used for subjective assessment of usability [14]. Each item has a scale position of 1 for strongly disagree, 2 for disagree, 3 for neutral, 4 for agree, and 5 for strongly agree. SUS scoring uses transformed data so that the final output can range from 0 to 100 representing the overall usability of the system under evaluation.

In this study, we surveyed KT Canada members, registered wiki members, and unregistered wiki visitors to gain a better understanding of our users, especially their experience with the wiki. The objective of this study is to gather the following information about the wiki: user’s perceptions, value, reasons for not engaging, suggestions to facilitate collaboration, and assessment of usability. The study was funded by KT Canada, which is an organization made up of Canadian researchers and educators working in the field of KT and is supported by funding from CIHR.

## Methods

### Web-Based Survey

To get a better idea of how and why people use the WhatisKT wiki, we surveyed three cohorts: KT Canada members, registered wiki members, and unregistered wiki visitors. A subset of survey participants agreed to a follow-up interview via Skype. The study received ethics approval from the McMaster Faculty of Health Sciences Research Ethics Board.

KT Canada members were invited to join the wiki soon after launch in 2008. Occasional email reminders of the wiki’s existence have been sent to this group since that time. This cohort was invited to take part in an online survey via the KT Canada weekly newsletter December 12, 2012. Survey participants were asked to fill in the same Web-based questionnaire. To obtain more in-depth information on user perceptions, the participants were also asked if they would be willing to volunteer for a virtual interview using Skype or another Web-based communication tool.

The wiki currently has over 115 registered members. This cohort was sent a link to the Web-based survey via the wiki interface on November 26, 2012, with a reminder sent on December 13, 2012.

The survey (Multimedia Appendix 1) included questions regarding their membership status, frequency of visits, experience with other wikis, and likelihood of collaboration on WhatisKT wiki. To understand user perception of the wiki, participants were asked to choose from a list of descriptors that describes the wiki: “a reference tool for KT terms”, “a collaboration platform”, and “a source of KT literature and links”. Participants also answered questions about barriers to engaging in the wiki and features that would make wiki collaboration easier. Respondents were not required to access the wiki during the survey. Only those participants who indicated they had visited the wiki completed the SUS questionnaire to assess the usability of the wiki and assessed content-related statements (Textbox 1) scored on a 5-point Likert scale. The score contributions for items 1, 3, 5, and 7 are calculated by deducting 1 from the scale position. The score contributions for items 2, 4, 6, and 8 are calculated by deducting the scale position from 5. The SUS score is 2.5 times the sum of scores of all 10 items [10]. Sauro reported an average SUS score across 500 usability evaluations with data from over 5000 users was 68 [15]. A SUS score <68 indicates below average usability, and a value equal or higher indicates above average usability. Descriptive statistics were performed on survey data.

**System Usability Scale (SUS) and content-related survey questions for KT Canada and wiki members.**

SUS items:

1. I think I would like to use this application frequently
2. I found the application unnecessarily complex
3. I thought the application was easy to use
4. I think I would need Tech Support help to use this application
5. I found the various functions in the application to be well integrated
6. I thought there was too much inconsistency in this application
7. I believe that most people would learn to use this application very quickly
8. I found the application very cumbersome to use
9. I felt very confident in using the application
10. I need to learn a lot about this application before I could effectively use it

Content-related items (not included in the SUS scoring):

1. It is easy to find the information that I need
2. The information provided by the wiki is easy to understand
3. The organization of information on the wiki is clear

### Interview

KT Canada or registered wiki members who volunteered to be interviewed were contacted and scheduled for a short, online interview. A standardized list of questions including membership status, frequency of visits, and involvement with other wikis was asked during the interview. Interviewees provided their overall impression about the wiki and a letter grade for the wiki. In addition, the participants were asked to provide three words to describe the wiki and comment on the best and least preferred characteristic of the wiki. Interviewees also commented on open-ended questions such as “what are your suggestions to improve the wiki?” and “If you were the wiki administrator, what would be the first thing you would do to improve the wiki?” The audio component of each interview session was recorded and transcribed. The planned analysis was a directed content analysis [16] performed by DM. Interview questions were targeted based on usability and wiki research with the intent to describe perceptions of the wiki, barriers and facilitators to contributing to the wiki, and usability of the interface. Interviews were transcribed and the data were grouped according to the key concepts of perceptions, barriers, facilitators, and possible improvements. Themes were developed within these concepts and supported by quotes from the interviewees. CL reviewed the content analysis with any disagreements resolved through consensus.

### Poll of Unregistered Visitors

Much of the wiki traffic is from unregistered visitors who come to the wiki via searches engines such as Google and from direct visits [11]. Short polls were displayed on a dedicated wiki page from January to March 2013 to capture responses from unregistered wiki visitors. The home page and highly accessed pages had prominent links to the poll page, which included two questions: (1) Which group describes you the best (researcher, clinician, decision/policy maker, educator, student, or other)? (2) What is the objective of your visit to the wiki (find definitions(s), find publication(s), learn more about KT, learn more about WhatisKT, none of the above)? Since our visitors tend to stay for a less than one minute, we opted to pose 2 short questions to get a sense of the types of unregistered visitors that view the wiki rather than limit responses with the burden of the longer survey.

## Results

### Web-Based Survey

Twenty-five KT Canada and registered wiki members began the Web-based survey over a period of approximately 50 days. Two sets of responses were incomplete and were excluded from analyses. Among the 23 respondents who completed the survey, most were researchers (Table 1) and 17 indicated that they had no prior experience using a wiki. The majority of the study participants became aware of WhatisKT wiki through KT Canada (n=12). Survey participants described the wiki as a reference tool (n=21), collaboration platform (n=10), and source of KT literature (n=9).

Sixteen participants indicated that they had used the wiki at least once and provided subjective usability assessment using the SUS questionnaire. The mean SUS score was 67 (95% CI 56-77), which was not statistically different from the average score of 68 reported from 500 usability evaluations [15] indicating that the wiki could be considered for design improvements. For content-related items, average score per question for ease of finding information was 3.69 (95% CI 3.67-3.70), ease of understanding information was 4 (CI 3.99-4.01), and clear organization of information was 3.63 (CI 3.61-3.64) out of a potential score of 5. Among the six participants who had previously collaborated on a wiki, only two visited WhatisKT wiki. They gave SUS scores of 97.5 and 60 and were not different in their ratings from non-wiki collaborators. Given the low number of responses, we were unable to look at subgroups of participants with and without wiki experience.

Fifteen participants indicated that they would likely collaborate on the wiki. However, two participants commented that time might be a significant barrier to collaboration. Other barriers included “complicated login” (n=1), “wiki edits are time consuming” (n=13), “lack of incentive” (n=3), “anxiety in editing the wiki” (n=4), and “need additional training” (n=4).

Participants indicated that “removal of user login” (n=5), “email notification regarding new terms or polls” (n=13), and “detailed tutorials” (n=8) could enhance collaboration. Six participants provided free-text comments on enhancing wiki collaboration such as “incentive to use wiki”, “requirement to use the wiki and protected time to do so”, and “email notifications on any new content such as new publications”.

Participants also indicated that they would like to see latest KT publications (n=17), discussion forums (n=10), user ratings for standardized KT terms (n=8), and more terms related to KT (n=5). Study participants indicated that wiki could be improved by providing email notification regarding new terms, better grouping of terms, user friendly interface, and training for users interested in editing the wiki.

**Table 1:** Demographic group of online survey and poll participants.

Demographic group	Survey participants (n=23)	Poll participants (n=10)
Researcher	10	6
Clinician	2	0
Decision or policy maker	0	1
Educator	1	0
Student	1	1
Other	9	2

### Interview

Six survey participants indicated a willingness to be interviewed but only 3, including a clinician and 2 researchers, returned follow-up emails and were interviewed via Skype. Two participants were wiki members. Two of the participants learned about the existence of WhatisKT wiki through word of mouth, and one participant found the wiki through online searching. The frequency of visits to the wiki ranged from couple of times since the wiki’s launch to a couple of times a month. Two participants visited or contributed to other wikis. Two participants were from Canada, and one was from the United States. Content analysis produced the following themes: reference resource, positive impressions with room for improvement, limitations, engaging contributors, and suggestions for improvements.

### Perceptions

All participants mentioned that the best characteristic of the wiki was the collection of definitions. It was seen as a valuable resource when writing manuscripts and for teaching purposes: “I find it very helpful to use as a reference point when I try to define terms in the KT/KM field to other people” and “So what is most useful for us is a collection of different definitions that also comes with a citable source”.

Overall, participants described the wiki as “linkable, helpful, and health focused”, “innovative, open, and complete”, and “informative, not as much used, and efficient”. A theme that emerged was that the wiki is viewed positively, but that it still needs work. Their overall impression of the wiki was positive, but when asked to rate the wiki they gave it a B or B– and 6 or 7 (out of 10). Comments included:

It is good—a great tool. It has a lot of visits. When you Google “Knowledge Translation” it is the first or second link that comes up.

B–because it answers the question, “What is KT?” But, may be if it was broadly used or had more diverse opinions or more discussion, I am not sure what is missing. I guess there is room for improvement. It is pretty good.

I think we really like it as a repository especially for different definitions. Where we disagree a bit—or where we think is too early—is that lot of the work that has been done on consensus and finding a definition that is agreed—because we think it is an evolving field.

My impression is that it is a wiki designed to collect, synthesize and create consensus about the definition of “What is KT”…and how people interpret the term implementation science, knowledge translation, knowledge-to-action and all sorts of varied terms that are used for the definition of implementation science and knowledge translation.

Participants mentioned that the Canadian perspective, health focus, and theoretical content were the least preferred characteristics of the wiki. The limitations in what the wiki provides emerged as a separate theme:

It’s more health focused than the work I am doing, so with sort of that lens in mind it is not always totally appropriate”, “…the definitions that are up there have a very Canadian flavor about them.

I consulted it in the past, I felt it a very interesting resource with many terms for KT, but beyond that I might not have had the time to push to analyze what the KT was used for. I didn’t feel any incentive to add more to what was already there.

### Possible Improvements

Participants suggested expanding the wiki’s content to include definitions from various disciplines and research communities. Another suggestion for improvement was to include practical KT tools. All three participants mentioned the need to engage the users and increase their participation in the wiki. However, two participants mentioned that lack of incentives might be a reason why members are not contributing to the wiki. These suggestions could be used in generating user interaction with the wiki in the future.

Trying to improve visibility, but also getting more content and more editing going on which involves forming active community. An interesting way to do it would be to have an ongoing cohort of graduate student class taking some sort of knowledge translation class curating the wiki which would keep it cleaned up.

What would be useful is to show difference and to show the different definitions that are out there—not a consensus—showing the variety of terms and variety in definitions.

### Barriers and Facilitators

Participants were specifically asked to describe barriers and facilitators for contributing to the wiki. Incentives, providing credit, developing critical mass, and defining a clear goal for the wiki emerged as ideas.

The reasons for not contributing: Not paid. We spend so much time worrying about full publication that we do not have the energy to contribute to another outlet.

People wondered—is there a critical mass involved that contribute to it? KT is an evolving field—too early to agree on definition.

I would try to see how I can engage the community that you are trying to get people to contribute to; to find the incentive that would encourage people to contribute to it, find ways of giving credits; continuing professional development credits for contributing to the wiki or ways of creating some sort of interest in getting people to contribute; recognizing contributions to it; ways of getting people to contribute to it.

Trying to engage the target knowledge users that you are aiming to get involved in the wiki so they understand where the [WhatisKT] wiki is going and what is the end goal of the wiki.

### Poll

Over the course of 2 months, we received only 10 responses from unregistered wiki visitors through the polls posted on the wiki. During that time, Google analytics tracked 2929 unique visitors, so the response rate was extremely low at 10/2929 (0.3%). Six respondents were researchers, 1 decision/policy maker, 2 students, and 2 identified as “other”. Participants indicated that the objective of their visit was to learn more about KT (n=6), learn more about WhatisKT (n=2), find definitions (n=1), and none of the above (n=1).

## Discussion

### Principal Results

In our Web-based survey, 16 participants provided a subjective assessment of the wiki’s usability via the SUS questionnaire, which provides the most reliable results with sample sizes of at least 12 to 14 participants [17]. The usability results from our study can be considered reliable despite the small sample size compared to typical clinical studies, though the confidence interval for the mean SUS score was fairly wide (56% to 77%). The online poll had only 10 responses over a period of 2 months, a response rate of only 0.3% of unique visitors. The low response rate is lower than studies involving recruitment of research participants through the Internet [18]. We had hoped to provide a pop-up poll to all unregistered visitors to maximize awareness of the poll and participation. Unfortunately, the wikispaces platform is not designed to accommodate such polls. Our small sample size for the polls, surveys, and interviews did not allow us to reach saturation, which is a primary limitation.

More researchers participated in both the online survey and the poll than any other demographic group. This could be an indication that researchers are prominent stakeholders interested in the wiki. The majority of the survey participants indicated that WhatisKT wiki could be best described as a reference tool, which was reiterated by interviewees. On the other hand, poll results indicate that the primary objective for some visitors is to learn more about KT and very few (10%) indicated that they were interested in the definitions, though with a sample size of 10, this observation is limited. These visitors could have reached the wiki through a search engine while searching for a KT-related term or concept. Such visitors could expect more KT-related content than just a repository of terms.

Despite the fact that one of the goals of our work was to understand the lack of uptake for the discussion function of the wiki, we were unable to resolve this issue. Although 10 respondents indicated an interest in discussion forums on the wiki, a functionality that is already available, no discussion has yet been started despite the wiki being 5 years old. This is not an unusual situation in collaborative writing projects, and frequently reported barriers include unfamiliarity and lack of skills with the technologies, time and work constraints, and concerns about quality of contributions, and legal ramifications [18]. Since participants indicated interest in these forums, we will continue to encourage and develop this function of the wiki. Barriers to contributing to discussion forums or content identified in the survey include time, issues related around the technology (complicated login and need for training), and lack of incentives. We will be considering how we can overcome some of these barriers.

Previous research has identified a number of potential facilitators such as training, ease of use of the system, having a moderator or champion to monitor the content and ensure quality, having a critical mass of content providers, creating a community of practice or learners, and providing incentives [5]. Currently, the wiki offers no incentive for edits although users are able to tag their contributions to identify themselves. For clinical members, continuing medical education credits could be provided as an incentive. For students, instructors could be encouraged to include wiki contributions as a required task, as was suggested by one of our interviewees. This guided interaction with the wiki could also generate the community of learners as a step towards reaching critical mass. For researchers, however, incentives and credit are more difficult to devise since their focus is on peer-reviewed publication and receiving grants. The specific facilitators or barriers to interactivity is something that requires further research and would benefit from moving beyond asking people if they are interested in interactivity to determining the specific conditions in which interactivity occurs.

Our study found that the wiki is being used as a reference tool. Survey participants were pleased with the wiki content and its quality. This was evident from the above average score given for organization, ease of finding, and ease of understanding the information provided on the wiki. However, the wiki users preferred better grouping of terms and having a friendly user interface. The term groupings, ”Core”, “Additional” and “Standardized” terms, could be re-organized into more intuitive groupings to facilitate ease of reference of terms and definitions in the KT domain. One limitation of wikis is that as the site is presented one page at a time, it can be difficult to get the overall picture of the wiki [19]. The wiki can be modified with easily accessible groupings of terms and an alphabetical list to provide a comprehensive view to all visitors.

Email notification regarding new terms or polls was mentioned as a feature that will enhance collaboration. In the future, it would be ideal to send wiki members email notifications when new terms or polls are added. Addition of latest KT publications can increase potential visits to the wiki.

### Limitations

Our primary limitation was the low response rate, which reduces the generalizability of the findings. This limitation demonstrates a common problem inherent in research in online tools [5]. However, this study comprises some of the first user-based knowledge summaries related to collaborative platforms. Given the growth in the use of these technologies, advancing what is known about optimizing these types of platforms is needed. By their nature, wikis present technical limitations for understanding “best wiki practices” through user engagement. We experienced technical limitations related to the wiki platform; our poll was included as a link on select pages in the wiki rather than a pop-up visible to all visitors. A pop-up containing the poll questions, for all visitors regardless of landing page, could have resulted in better response rate from wiki visitors. Additionally, since we anticipated fewer response rates from unregistered wiki visitors and were concerned about burden, we posted only two questions. A more elaborate poll could have probed into the specific needs and usability concerns of non-registered visitors but at the potential risk of response rate. Improved understanding of weighing these issues in online data collection is needed. Due to the nature of using a commercial wiki platform, we are unable to describe our cohort. This further limits our ability to contextually analyze the data that we collect. The present study described the usability assessment from KT Canada and registered wiki members. The general wiki visitor was not provided the opportunity for usability assessment. An elaborate survey targeting all wiki visitors could give an indication of the issues and needs of the general wiki visitor.

### Future Work

In response to this survey, enhancements have already been made to the wiki. For example, we have regrouped our KT terms into categories: KT science terms, KT intervention terms, KT tools and methods, and miscellaneous terms based on consensus within our team. KT science terms describe or define the process of (1) having a body of evidence, information, knowledge, research and (2) moving it into practice, application, use. KT intervention terms describe or define a method or set of methods that will be done to enable (an act of intervening) a body of evidence, information, knowledge, research to be moved into practice. Intervention term examples are audit and feedback, communities of practice, and opinion leader/educational influential. KT tools and methods terms describe or define a tool or technique that aids the whole KT process or a part of the KT process. For example, tool terms are gap analysis, knowledge synthesis, and comparative effectiveness research. Miscellaneous KT terms are used in KT context, but not included in the categories above.

However, content changes will not be enough to improve activity on the wiki. We will look at the previously reported facilitators and determine which might be best to generate contributions to the wiki. We have started discussing ways to facilitate improved interaction with the wiki, including conversations with graduate course providers, but these actions have not yet been implemented. Also, by having a dedicated staff person to moderate and verify any new content, we can ensure the quality of the information used to build the wiki.

### Conclusions

This study has shown that WhatisKT wiki is being used mainly as a reference tool by users. The wiki scored reasonable for usability, but study participants indicated a number of barriers and facilitators to adding content and contributing to discussion forums. We will focus our efforts on improving the usability and testing strategies to remove barriers.

## Multimedia Appendix 1

Web survey completed by KT Canada members and registered wiki members.

## Abbreviations

CIHR: Canadian Institutes of Health Research
KT: knowledge translation
