# Supplementary material for: Engaging With a Wiki Related to Knowledge Translation: A Survey of WhatisKT Wiki Users
Source: J Med Internet Res. 2014 Jan 21;16(1):e21. doi: 10.2196/jmir.3001 (PMC3906698; doi:10.2196/jmir.3001)
Supplement: Supplementary file 1 [file jmir_v16i1e21_app1.pdf]

WhatisKT_Survey

The whatisKT wiki has been online since 2008 at http://whatiskt.wikispaces.com/. The wiki is a compendium of terms and definitions related to Knowledge translation (KT). The wiki was designed to be a collaborative platform for KT researchers and stakeholders to debate the use of KT-related terms and their usage. Over the past 4 years the wiki has been visited regularly by people all over the world. However, visitors tend to visit only a few pages, and, to date, no user has contributed source material or started a discussion.

Objectives:Our intent with this study is to survey whatisKT wiki visitors, members and nonmembers to gain a better understanding of who our visitors are, what value they get from the wiki, why they have not engaged in the wiki, and ways we can facilitate collaboration on the wiki.
Thank you for taking the time to take our survey on WhatisKT wiki. The survey will take approximately 5 to 10 minutes of your time. Your feedback will help us improve the usefulness and usability of the wiki.This research study has received ethics approval from the McMaster Faculty of Health Sciences Research Ethics Board. Submission of your survey responses is an indication of your consent to take part in the study.If you have any questions please contact Cynthia Lokker, Research Coordinator at lokkerc@mcmaster.ca or 905-525-9140 x22208. All mandatory questions are indicated with * in front. You can leave the study at any time.

There are 17 questions in this survey

Demographics

1 [D01]Please indicate below the group that best describes you. *

Please choose **only one** of the following:

- ◯ Researcher
- ◯ Clinician
- ◯ Decision Maker
- ◯ Policy Maker
- ◯ Educator
- ◯ Student
- ◯ Other [ ]

2 [D02]Please indicate your membership status. *

Please choose **only one** of the following:

- ◯ WhatisKT wiki member
- ◯ KT Canada member
- ◯ Member of both WhatisKT wiki and KT Canada
- ◯ Other [ ]

KTCanada

3 [KT01]Were you aware of the WhatisKT wiki? *

Only answer this question if the following conditions are met:
° Answer was 'KT Canada member' at question '2 [D02]' (Please indicate your membership status.)

Please choose **only one** of the following:

- Yes
- No

4 [KT02]Have you joined WhatisKT wiki? *

Only answer this question if the following conditions are met:
° Answer was 'KT Canada member' at question '2 [D02]' (Please indicate your membership status.)

Please choose **only one** of the following:

- Yes. Why?
- No. Why not?

Make a comment on your choice here:

WhatisKT

5 [W01]Why did you join the wiki?

Only answer this question if the following conditions are met:
° Answer was 'WhatisKT wiki member' or 'Member of both WhatisKT wiki and KT Canada' at question '2 [D02]' (Please indicate your membership status.)

Please choose **all** that apply:

- ☐ I was invited to join WhatisKT wiki
- ☐ I thought WhatisKT wiki is a good platform for collaboration
- ☐ I wanted to participate in standardizing KT terms
- ☐ I wanted to edit WhatisKT wiki contents
- ☐ Other: ______

Wiki Collaboration

6 [WC01A]How did you come to know of WhatisKT wiki? *

Please choose **only one** of the following:

- Through KT Canada
- Through McMaster University
- Through a Search Engine (Google, Bing, etc.)
- Through a WhatisKT wiki member/user
- Other

7 [WC01]In your opinion which of the following options describes the WhatisKT wiki? *

Please choose **all** that apply:

- A reference tool for KT terms and definitions
- A collaboration platform
- A source of KT literature and links
- Other:

8 [WC02]How often do you visit WhatisKT wiki? *

Please choose **only one** of the following:

- Daily
- Few times in the past 7 days
- Few times in the past 30 days
- Few times in the past 12 months
- Few times since the wiki started
- Never

9 [WC03]Have you ever collaborated on a wiki other than WhatisKT? *

Please choose **only one** of the following:

- Yes
- No

10 [WC04]Would you be likely to collaborate on this wiki? *

Please choose **only one** of the following:

- ○ Yes.
- ○ No. Why not?

Make a comment on your choice here:

11 [WC05]What would make collaboration easier for you?

Please choose **all** that apply:

- ☐ Removal of user login to edit the wiki
- ☐ Email notification regarding new terms or polls
- ☐ Detailed tutorials on using the wiki
- ☐ Other:

12 [WC06]What are the barriers to engaging in WhatisKT wiki?

Please choose **all** that apply:

- ☐ The wiki is difficult to edit
- ☐ The login to the wiki is complicated
- ☐ Editing the wiki made me feel nervous
- ☐ Engaging in the wiki is time consuming
- ☐ There is no incentive for participating in the wiki
- ☐ I need additional training to engage in the wiki
- ☐ Other:

13 [WC07]How could WhatisKT wiki serve you better?

Please choose **all** that apply:

- ☐ Provide better grouping of KT terms
- ☐ Email members when a new term or definition is posted
- ☐ Make the interface more user friendly
- ☐ Provide additional training for users interested in editing the wiki
- ☐ Other: ______

14 [WC08]What other information would you like to see on the wiki?

Please choose **all** that apply:

- ☐ Discussion Forum
- ☐ Latest KT Publications
- ☐ More terms related to KT
- ☐ User rating for standardized KT terms
- ☐ Other: ______

Wiki Usability and Content

15 [SUS_items]Following 10 items are regarding the usability of WhatisKT wiki. Please indicate your response for ALL items. 1=Strongly Disagree, 2=Disagree, 3=Neutral, 4=Agree, 5=Strongly Agree *

Only answer this question if the following conditions are met:
° Answer was NOT 'Never' at question '8 [WC02]' (How often do you visit WhatisKT wiki?)

Please choose the appropriate response for each item:

	1	2	3	4	5
I think that I would like to use this wiki frequently	○	○	○	○	○
I found the wiki unnecessarily complex	○	○	○	○	○
I thought the wiki was easy to use	○	○	○	○	○
I think that I would need the support of a technical person to be able to use this wiki	○	○	○	○	○
I found the various functions in this wiki were well integrated	○	○	○	○	○
I thought there was too much inconsistency in this wiki	○	○	○	○	○
I would imagine that most people would learn to use this wiki very quickly	○	○	○	○	○
I found the wiki very cumbersome to use	○	○	○	○	○
I felt very confident using the wiki	○	○	○	○	○
I needed to learn a lot of things before I could get going with this wiki	○	○	○	○	○

16 [CSUQ_content]Following 3 items are regarding the content of WhatisKT wiki. Please indicate your response for ALL items. 1=Strongly Disagree, 2=Disagree, 3=Neutral, 4=Agree, 5=Strongly Agree *

Only answer this question if the following conditions are met:
° Answer was NOT 'Never' at question '8 [WC02]' (How often do you visit WhatisKT wiki?)

Please choose the appropriate response for each item:

	1	2	3	4	5
It is easy to find the information that I need.	○	○	○	○	○
The information provided by the wiki is easy to understand.	○	○	○	○	○
The organization of information on the wiki is clear.	○	○	○	○	○

Interview

17 [I01]Would you be willing to be interviewed ? *

Please choose **only one** of the following:

- No
- Yes. Please provide your email for scheduling an interview

Thank you for taking the time to take our survey on WhatisKT wiki. If you have any questions about this survey please contact Cynthia Lokker, Research Coordinator at lokkerc@mcmaster.ca or 905-525-9140 x22208.
31.12.1969 – 19:00

Submit your survey.
Thank you for completing this survey.
